# Targeted Vestibular Rehabilitation Accelerates Symptom Relief by Promoting Vestibular Compensation in BPPV Patients With Residual Symptoms: Evidence From Resting‐State fMRI

**DOI:** 10.1002/brb3.71696

**Published:** 2026-08-20

**Authors:** Gendi Yin, Yinfei Liang, Qiling Huang, Lingwei Li, Jiashu Lin, Chang Liu, Zhicheng Li, Xiangli Zeng

**Affiliations:** ^1^ Department of Otolaryngology‐Head & Neck Surgery The Third Affiliated Hospital of Sun Yat‐Sen University Guangzhou China; ^2^ Center of Dizziness and Tinnitus Diagnosis and Treatment The Third Affiliated Hospital of Sun Yat‐Sen University Guangzhou China

**Keywords:** benign paroxysmal positional vertigo, residual dizziness (RD), targeted vestibular rehabilitation therapy (tVRT)

## Abstract

**Aims:**

Residual dizziness (RD) is commonly observed in patients with benign paroxysmal positional vertigo (BPPV) following successful canalith repositioning procedures. Targeted vestibular rehabilitation therapy (tVRT) is a novel strategy designed to address specific vestibular deficits. This study aims to investigate whether tVRT can accelerate the vestibular rehabilitation process in patients with RD of BPPV and explore its neural mechanisms.

**Methods:**

Ninety‐four BPPV patients with RD were enrolled and received a 4‐week tVRT program tailored to utricular deficits. A rs‐fMRI subgroup (*n* = 43) was used to assess brain activity and functional connectivity (FC) before and after successful intervention.

**Results:**

The tVRT group demonstrated significantly lower DHI and SAS scores and higher ABC score. rs‐fMRI revealed that tVRT increased ALFF in the right superior temporal gyrus (STG), positively correlated with ABC scores, and enhanced ReHo in the right anterior cingulate cortex (ACC). The tVRT group showed enhanced STG‐cerebellar tonsil connectivity, along with reduced STG‐posterior cingulate cortex connectivity. Using right ACC as seed, FC between the ACC and lingual gyrus was increased. STG–cerebellar tonsil FC was negatively correlated with RD duration.

**Conclusion:**

tVRT accelerates vestibular compensation in patients, which may be mediated by enhanced neural activity and reorganization of brain networks involved in vestibular processing.

## Graphical abstract

Targeted vestibular rehabilitation therapy does not improve utricular function directly, but improve dizziness, anxiety, and balance confidence to accelerate vestibular compensation, by enhanceing brain functional plasticity

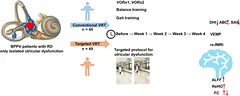

**Abbreviations**
ABC: activities‐specific balance confidence
ACC: anterior cingulate cortex
ALFF: amplitude of low‐frequency fluctuation
BOLD: blood oxygenation level‐dependent
BPPV: benign paroxysmal positional vertigo
CRP: canalith repositioning procedure
CT: caloric testing
cVEMP: cervical vestibular‐evoked myogenic potentials
DHI: Dizziness Handicap Inventory
DMN: default mode network
EPI: echo‐planar imaging
FC: functional connectivity
FOV: field of view
KCC: Kendall's coefficient of concordance
MNI: Montreal Neurological Institute
oVEMP: ocular vestibular‐evoked myogenic potentials
RD: residual dizziness
ReHo: regional homogeneity
REML: mixed‐effects model
rs‐fMRI: resting‐state functional magnetic resonance imaging
SAS: Self‐Rating Anxiety Scale
STG: superior temporal gyrus
SVV: subjective visual verticality
TE: echo time
TR: repetition time
tVRT: targeted vestibular rehabilitation therapy
vHIT: video head impulse test
VOR: vestibulo‐ocular reflex
VRT: vestibular rehabilitation therapy

## 1 Introduction

Benign paroxysmal positional vertigo (BPPV) is one of the most common peripheral vestibular disorders. The canalith repositioning procedure (CRP) remains the most widely used and well‐accepted first‐line treatment for BPPV, owing to its simplicity, safety, and effectiveness (Kim et al. 2021). However, despite its clinical efficacy, CRP has notable limitations. A significant proportion of patients experience residual dizziness (RD) following successful repositioning, the incidence of RD has been reported to range from 31% to 61% (Seo et al. 2017; Vaduva et al. 2018). RD is characterized by nonspecific symptoms such as light‐headedness, fogginess, or unsteadiness, persisting from several days to as long as 2 months, with some cases progressing to chronic symptoms (Özgirgin et al. 2024). Therefore, there is an urgent need for more effective therapeutic strategies to alleviate dizziness in BPPV patients with RD and improve their quality of life.

Notably, accumulating clinical evidence suggests that utricular dysfunction plays a more prominent role than saccular dysfunction in the development of RD. Studies have demonstrated that patients with RD often exhibit more severe utricular impairment compared to those without RD, indicating a crucial contribution of utricular deficits to the persistence of post‐repositioning symptoms (Seo et al. 2017). Ocular vestibular‐evoked myogenic potentials (oVEMP) are neurophysiological tests that assess the integrity of the utriculo–superior vestibular nerve pathway by recording evoked myogenic responses from extraocular muscles. Compared with cervical vestibular‐evoked myogenic potentials (cVEMP), which primarily reflect saccular function, oVEMP are more sensitive indicators of utricular dysfunction. Therefore, patients presenting with isolated oVEMP abnormalities can be considered to exhibit a distinct phenotype of utricular‐specific vestibular impairment. Studies have reported that the prevalence of abnormal oVEMP responses in patients with BPPV is approximately 57.2%, significantly higher than that in healthy controls (Niu et al. 2022). This finding suggests that the pathophysiology of BPPV may involve not only otoconial dislodgement but also bilateral degeneration of otolithic organs, particularly the utricle. In patients with BPPV who show isolated oVEMP abnormalities, the clinical relevance of residual dizziness is especially pronounced. Notably, oVEMP abnormalities have been associated with an increased risk of BPPV recurrence (López‐Viñas et al. 2024; Rosa et al. 2023), and some studies propose that abnormal oVEMP findings may serve as a predictive marker for recurrence (Kunelskaya et al. 2023). Hoseinabadi et al. further demonstrated that utricular dysfunction significantly increases the likelihood of BPPV recurrence. Importantly, vestibular rehabilitation programs that combine habituation training with targeted exercises for utricular function have been shown to reduce recurrence rates effectively (Hoseinabadi et al. 2018). These findings suggest that utricular dysfunction not only serves as a valuable predictor of BPPV recurrence but also provides a rational basis for tailoring individualized and effective vestibular rehabilitation strategies.

To facilitate vestibular compensation, vestibular rehabilitation therapy (VRT) is commonly recommended as an adjunctive intervention following CRP in BPPV patients with persistent RD. Conventional VRT typically consists of gaze stability exercises to enhance vestibulo‐ocular reflex (VOR) function, together with balance and gait training to promote vestibulospinal compensation and postural control. Studies have demonstrated that VRT, which facilitates vestibular compensation and behavioral adaptation, is a safe and effective approach for managing unilateral peripheral vestibular dysfunction (Hillier and McDonnell 2011). Although vestibular compensation induced by such rehabilitation typically occurs naturally in most individuals and proves beneficial for alleviating residual symptoms, the duration of compensation varies considerably across patients (Lacour et al. 2016), and therapeutic effects are often delayed. Therefore, accelerating the process of vestibular recovery remains a critical challenge that urgently needs to be addressed.

In this study, we propose for the first time an innovative concept of targeted vestibular rehabilitation therapy (tVRT), which aims to precisely address the site of vestibular dysfunction in patients with dizziness. The goal of tVRT is to accelerate the recovery process in BPPV patients with RD, thereby improving symptoms of vertigo and imbalance. To date, no studies have been published on this targeted rehabilitation approach. This study aims to evaluate the effectiveness of tVRT in improving balance function and reducing recovery time in BPPV patients exhibiting isolated utricular dysfunction. Outcomes will be compared with those of conventional vestibular rehabilitation, with the ultimate aim of providing clinical evidence to support optimized, individualized strategies for accelerating vestibular compensation.

## 2 Materials and Methods

### 2.1 Participants

This study enrolled patients diagnosed with BPPV‐related RD associated with isolated utricular dysfunction, based on the update 2008 Clinical Practice Guideline for the Diagnosis and Treatment of BPPV (Bhattacharyya et al. 2017). A total of 104 patients who visited the Department of Otolaryngology‐Head and Neck Surgery at the Third Affiliated Hospital of Sun Yat‐Sen University between January 2019 and December 2024 were initially recruited. After excluding 10 patients due to loss to follow‐up and other reasons, 94 eligible patients were ultimately included in the study.

All participants underwent comprehensive clinical evaluations, including detailed medical history, physical examination, audiological assessment, vestibular function testing, and neuroimaging. Vestibular function was assessed using caloric testing (CT), video head impulse test (vHIT), oVEMP, cVEMP, and subjective visual verticality (SVV).

Inclusion criteria: age between 18 and 60 years; diagnosis of RD secondary to BPPV with evidence of isolated utricular dysfunction confirmed by vestibular function tests (normal CT, vHIT, cVEMP, and abnormal oVEMP and/or SVV).

Exclusion criteria: diagnosis of Meniere's disease, sudden sensorineural hearing loss, autoimmune diseases, hereditary disorders, acute labyrinthitis, traumatic or space‐occupying lesions, or vertigo clearly attributed to ischemic causes; presence of severe neurological or cardiovascular diseases, including coronary artery disease, myocardial infarction, or stroke; inability to complete VRT due to physical limitations (e.g., mobility impairment); secondary hearing loss; subjects experiencing recurrent BPPV episodes during the observation period.

A total of 104 patients were randomly assigned using a random number table to either the conventional vestibular rehabilitation treatment group (*n* = 52, control group) or the tVRT group (*n* = 52). During follow‐up, 10 patients were lost to follow‐up and subsequently excluded. The final analysis included 94 patients who completed the clinical follow‐up were included in the clinical outcome analysis, comprising 45 in the control group and 49 in the tVRT group.

### 2.2 Criteria for Identifying Isolated Utricular Dysfunction

The diagnosis of isolated utricular dysfunction was determined based on the combined results of oVEMP, cVEMP, and SVV. An abnormal oVEMP was defined as either the absence of an identifiable N1–P1 waveform or an interaural amplitude asymmetry ratio >30%. An abnormal cVEMP was defined as either the absence of an identifiable P1–N1 waveform or an interaural amplitude asymmetry ratio >30%. Isolated utricular dysfunction was diagnosed when abnormal oVEMP findings were present in conjunction with normal cVEMP findings. For patients with initial findings suggestive of utricular impairment, indicated by isolated oVEMP abnormalities, a two‐step confirmation process was performed. First, neurophysiological verification was conducted by repeating VEMP testing on the day following the initial assessment. oVEMP was re‐examined and cVEMP was simultaneously reviewed to confirm the lesion specificity. To ensure the reliability of results, potential confounding factors such as patient cooperation (e.g., posture maintenance), environmental interference (e.g., electromagnetic noise, audiovisual stimuli), and technical issues (e.g., electrode impedance, stimulus parameters) were carefully controlled. Second, spatial orientation function was assessed using a standardized SVV testing system. At least 10 valid measurements were obtained and averaged, with a deviation of more than 2° considered a positive indicator. Patients meeting both criteria were diagnosed as having isolated utricular dysfunction.

After confirmation and enrollment, the presence and duration of RD were evaluated using a structured questionnaire. Patients were first asked whether they still experienced spinning sensations during head turning or rising after canalith repositioning procedure (CRP). If the answer was “yes,” incomplete repositioning was suspected and further CRP was recommended. If the answer was “no,” patients were asked whether they experienced dizziness, discomfort, imbalance, floating sensations, or neck tightness after CRP. An affirmative response was considered indicative of RD, and the duration of RD was recorded during follow‐up.

### 2.3 Vestibular Rehabilitation Therapy Strategy

All patients were hospitalized for 5–7 days and received standardized medical therapy during their stay, including intravenous injection of Ginkgo biloba extract (25 mL, once daily), and oral administration of betahistine mesylate (12 mg, three times daily) and mecobalamin (0.5 mg, once daily). Upon discharge, patients continued oral treatment with betahistine (12 mg, three times daily) and mecobalamin (0.5 mg, once daily) for 1 month. Vestibular rehabilitation was initiated on the first day after successful canalith repositioning, once residual dizziness was confirmed, and this day was defined as Day 1 of the rehabilitation program. All participants completed the same 4‐week rehabilitation protocol regardless of hospitalization length. During hospitalization, training was supervised by therapists, whereas after discharge the same standardized program was continued at home.

VRT was performed daily as follows: For control group, patients underwent standard conventional rehabilitation program, including gaze stabilization exercises, balance training, and gait training (rehabilitation protocols 1+2+3). The exercises were performed three times daily, with 30 repetitions per activity, lasting approximately 3–12 min per session. For tVRT group: In addition to the conventional rehabilitation program, patients received targeted vestibular rehabilitation that included variable‐speed lateral and anteroposterior translation training (rehabilitation protocols 1+2+3+4). Training frequency and duration were identical to those in the conventional group (three sessions daily, 30 repetitions per activity, approximately 3–12 min per session).

**FIGURE 1:**
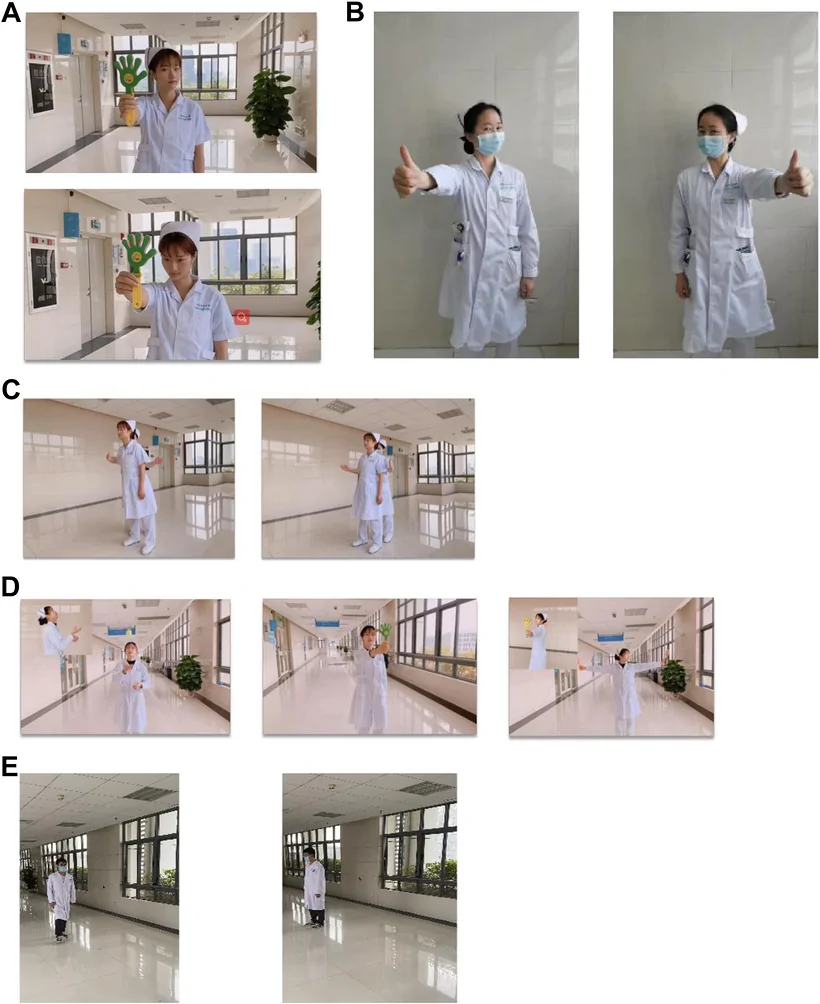
**Diagram of vestibular rehabilitation therapy**. (A) The images depict VORx1 exercises of Protocol 1. (B) The images depict VORx2 exercises of Protocol 1. (C) The images depict balance training of Protocol 2. (D) The images depict gait training of Protocol 3. (E) The images targeted training for utricular dysfunction of Protocol 4.

Protocol 1: Gaze stability exercises. During head movements in both the horizontal and vertical planes, participants were required to maintain constant visual fixation on a target and ensure its clarity throughout the motion. In the VORx1 exercise (Figure 1A), patients performed head oscillations along the yaw (horizontal) and pitch (sagittal) planes while fixating on a stationary target. In the VORx2 exercise (Figure 1B), patients were instructed to fixate on a target while both the head and the target moved simultaneously with equal amplitude but in opposite directions.

Protocol 2: Balance training. Participants performed anteroposterior and mediolateral weight‐shifting exercises under the supervision of trained personnel to prevent falls (Figure 1C).

Protocol 3: Gait training. Participants performed walking‐based exercises that included ball‐throwing tasks, visual fixation on a single hand‐held target, and alternating gaze fixation between targets held in both hands (Figure 1D).

Protocol 4: Targeted protocol for utricular dysfunction. In the upright position, the utricle detects linear acceleration in the interaural (mediolateral) and anteroposterior directions. In cases of unilateral vestibular dysfunction, an imbalance arises in the afferent signals transmitted from the bilateral otolith organs. Based on the principle of post‐lesional rebalancing, customized rehabilitation training involving variable‐speed translational movements in the interaural and anteroposterior directions is designed to specifically target the impaired region (Figure 1E). Speed range: Initial stage (week 1): 0.2–0.5 m/s; progressive stage (weeks 2–4): 0.5–1.0 m/s, adjusted according to patient tolerance. Training intensity: Each session includes 3 sets of 30 repetitions for both lateral and anteroposterior directions, with 30‐s rest between sets. Progression criteria: Increase speed by 0.1 m/s when patients can complete the current intensity without dizziness/VAS score increase ≥2 points. Safety monitoring: A trained therapist stays beside the patient throughout the training to prevent falls; training is terminated immediately if the patient reports severe vertigo, nausea, or chest tightness.

### 2.4 Resting‐State Functional Magnetic Resonance Imaging (Rs‐fMRI) Analysis

The rs‐fMRI was conducted to investigate brain functional alterations following 4 weeks of vestibular rehabilitation in both the conventional rehabilitation (control) group and the tVRT group. The rs‐fMRI analysis was designed as a pre‐specified exploratory neuroimaging subgroup study. Only participants who completed both baseline and post‐treatment MRI examinations were included in the imaging analysis.

Patients with contraindications to MRI (e.g., metallic implants, claustrophobia) were excluded prior to scanning. Imaging was performed using a 3.0 Tesla MRI system (ARCHITECT, GE Healthcare, USA) equipped with a 48‐channel head coil. High‐resolution structural images were acquired using a 3D‐T1 BRAVO sequence with the following parameters: repetition time (TR) = 7.7 ms, echo time (TE) = 3.1 ms, flip angle = 12°, field of view (FOV) = 256 × 256 mm^2^, matrix = 256 × 256, slice thickness = 1 mm with no interslice gap, 180 slices in total, and a total scan time of 4 min and 18 s. Functional images were acquired using a gradient echo‐planar imaging (EPI) sequence sensitive to blood oxygenation level‐dependent (BOLD) contrast, with parameters as follows: TR = 2000 ms, TE = 35 ms, flip angle = 90°, FOV = 240 × 240 mm^2^, matrix = 64 × 64, slice thickness = 4 mm with no interslice gap, 38 axial slices, 250 time points, and a total acquisition time of 8 min and 20 s.

During the scan, participants were instructed to lie supine with their heads immobilized to minimize motion artifacts. All participants were asked to remain awake, relaxed, keep their eyes closed, and refrain from engaging in any specific cognitive tasks. High‐resolution T1‐weighted structural images were collected for anatomical localization and spatial normalization during post‐processing.

Data preprocessing and analysis were performed using MATLAB 2023a with the DPABI toolbox (http://rfmri.org/dpabi) built upon SPM (Statistical Parametric Mapping, https://www.fil.ion.ucl.ac.uk/spm/). Standard preprocessing steps included slice timing correction, realignment, normalization to Montreal Neurological Institute (MNI) template, and spatial smoothing. Amplitude of low‐frequency fluctuation (ALFF) was computed to assess regional spontaneous neural activity. Regional homogeneity (ReHo) was calculated using Kendall's coefficient of concordance (KCC) to evaluate the synchronization of each voxel's time series with its neighboring voxels (6, 18, or 26 adjacent voxels). ReHo maps were subsequently standardized using Z‐transformation to reduce inter‐subject variability. Functional connectivity (FC) was analyzed using a seed‐based approach. A predefined brain region or voxel was selected as the seed, and its time series was extracted. Pearson's correlation coefficients were computed between the seed time series and the time series of all other voxels or regions of interest in the brain. The resulting correlation maps were transformed into Z‐scores using Fisher's *r*‐to‐*z* transformation and used for subsequent statistical analyses. All voxel‐wise statistical maps were corrected for multiple comparisons using Gaussian random field theory (GRFT) via the AlphaSim procedure implemented in DPABI. The cluster‐forming threshold was set at *p* < 0.001 (uncorrected) at the voxel level, combined with a cluster‐level threshold of *p* < 0.05 (corrected) to define significant clusters. The minimum cluster size was 20 voxels for all ALFF, ReHo, and FC analyses.

### 2.5 Outcome Measures

Prior to treatment, all participants underwent baseline assessments including vHIT, cVEMP, oVEMP, and CT. Following group assignment, participants were re‐evaluated at 1, 2, 3, and 4 weeks after initiation of vestibular rehabilitation. The reassessments included vHIT, cVEMP, and oVEMP. Additionally, the Activities‐specific Balance Confidence (ABC) scale, the Chinese version of the Dizziness Handicap Inventory (DHI), and the Self‐Rating Anxiety Scale (SAS) were administered to evaluate balance confidence, dizziness‐related disability, and anxiety levels, respectively, at 1, 2, 3, and 4 weeks after initiation of vestibular rehabilitation.

### 2.6 Blinding

Owing to the nature of the rehabilitation intervention, participants and therapists could not be blinded to group allocation. However, all questionnaire‐based outcomes (DHI, ABC, and SAS) were assessed by the same physician, who was blinded to treatment allocation and was not involved in patient randomization or rehabilitation delivery. Vestibular function tests (oVEMP, cVEMP), vHIT, and rs‐fMRI were also performed by a separate examiner who was blinded to treatment allocation. Statistical analyses were conducted by an independent investigator blinded to group allocation.

### 2.7 Statistical Analysis

All statistical analyses were performed using GraphPad Prism version 10.1.2 (GraphPad Software, San Diego, CA, USA). Continuous variables were first tested for normality and homogeneity of variances. For data meeting the assumptions of normal distribution and equal variances, between‐group comparisons were performed using independent‐samples *t*‐tests. Longitudinal repeated measurements were analyzed using a mixed‐effects model (REML) with fixed effects for group, time, and the group × time interaction. Because some follow‐up data were missing, the mixed‐effects model was used instead of repeated‐measures ANOVA. Geisser–Greenhouse correction was applied when appropriate. When a significant group × time interaction was detected, post hoc simple‐effects analyses were performed to compare the two groups at each time point using Šidák's multiple comparisons test. Within‐group comparisons across time were additionally performed using Tukey's multiple comparisons test. For data that did not meet normality or homogeneity assumptions, non‐parametric tests were used, with the Mann–Whitney *U* test applied for between‐group comparisons. Categorical variables were compared using the chi‐square test, conducted in R software (version 3.5.3). A *p*‐value of less than 0.05 was considered statistically significant.

## 3 Results

### 3.1 Baseline Characteristics of BPPV Patients With RD

A total of 104 patients diagnosed with RD following BPPV and confirmed to have isolated utricular dysfunction were initially enrolled in this study. After excluding 10 patients due to loss to follow‐up or other reasons, 94 patients were ultimately included in the final clinical outcome analysis (Table S1). Among them, 45 patients were assigned to the conventional rehabilitation control group (23 males and 22 females; mean age: 47.33 ± 8.72 years), and 49 patients were assigned to the tVRT group (25 males and 24 females; mean age: 48.09 ± 10.20 years). There were no statistically significant differences between the two groups in terms of age or sex. In addition, comparison of baseline scale scores revealed no significant differences between the two groups in DHI, SAS, or ABC scores, indicating comparable initial clinical status between the groups.

Overall adherence to the rehabilitation protocol was high. A total of 87 of 94 patients (92.6%) completed at least 90% of the prescribed training sessions, with no significant difference between the conventional and tVRT groups. The primary reason for reduced adherence was transient training‐related dizziness, which resolved spontaneously within 1 week. No serious adverse events, including falls, syncope, or cardiovascular events, were observed during the intervention period.

### 3.2 TVRT Does Not Accelerate the Recovery of Utricular Function in BPPV Patients With RD

**FIGURE 2:**
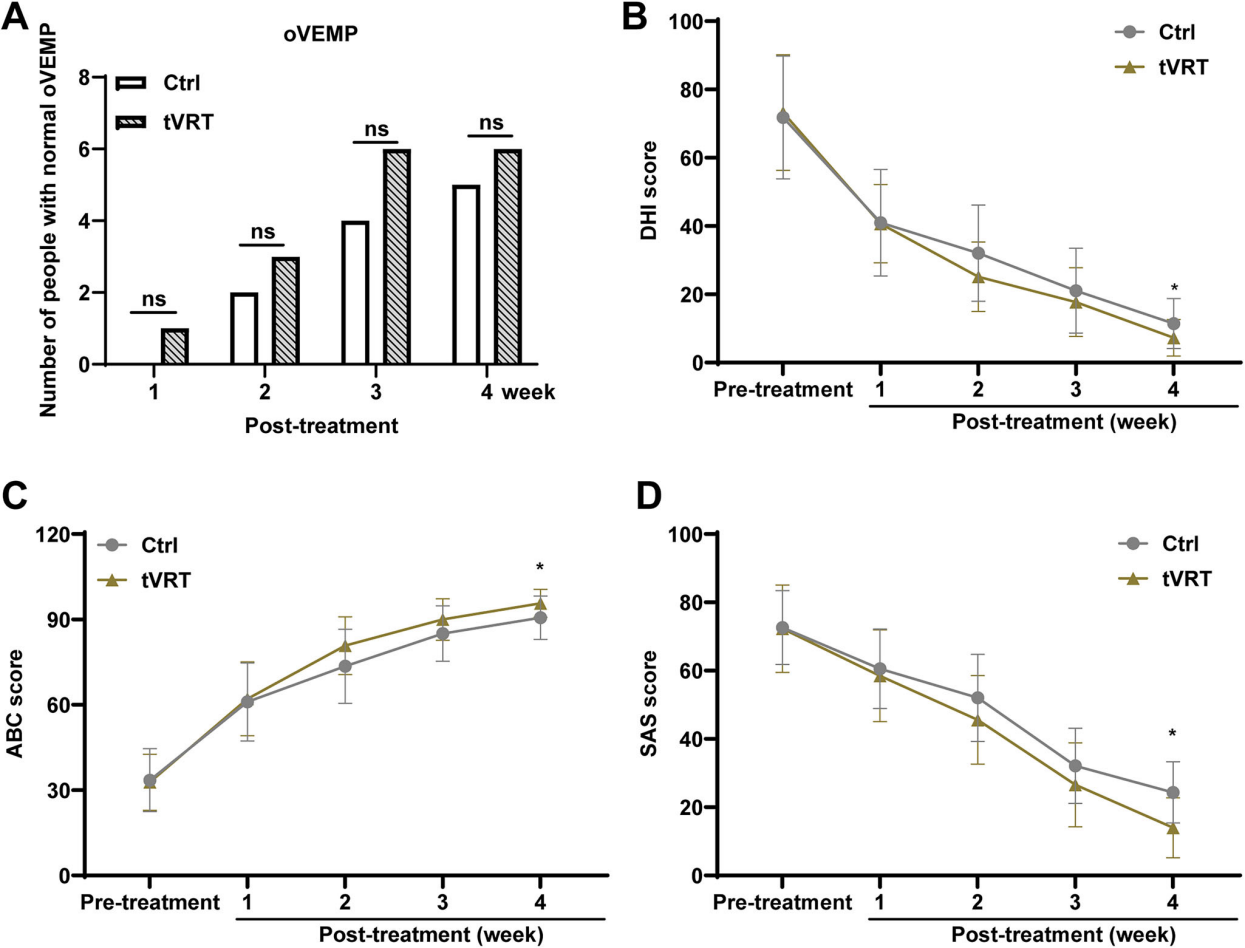
**tVRT accelerates improvement in symptom severity scores in BPPV Patients with RD**. (A) Comparison chart of oVEMP response of patients in the two groups at different periods. Fisher's exact test. ns *p* > 0.05 versus the control group. (B) Comparison chart of DHI scores of patients in the two groups at different periods. (C) Comparison chart of ABC scores of patients in the two groups at different periods. (D) Comparison chart of SAS scores of patients in the two groups at different periods. (B–D) Data are presented as mean ± SD. Statistical analysis was performed using a mixed‐effects model (REML) with fixed effects for group, time, and the group × time interaction. Post hoc between‐group comparisons at each time point were performed using Šidák's multiple comparisons test. *p < 0.05 versus the control group.

To evaluate changes in vestibular otolith organ function, cVEMP and oVEMP responses were assessed before and after treatment. As shown in Table S2 and Figure 2A, cVEMP results, reflecting saccular function, exhibited no significant changes following intervention in either group. oVEMP results, which indicate utricular function, demonstrated a gradual improvement over time in both the control and tVRT groups. However, no significant differences were observed between the two groups at any time point, suggesting that compared to conventional vestibular rehabilitation, tVRT does not accelerate the recovery of utricular function in BPPV patients with residual dizziness.

### 3.3 TVRT Targeting the Utricle Does Not Affect Semicircular Canal Function in BPPV RD Patients With Isolated Utricular Dysfunction

To assess the impact of tVRT on semicircular canal function, vHIT and CT were performed. Since BPPV patients only with isolated utricular dysfunction were enrolled, their semicircular canal function was normal at baseline. As shown in Table S3, there were no significant differences in the abnormality rates of semicircular canal function between the control and tVRT groups before or after treatment, indicating that tVRT targeting the utricle does not influence semicircular canal function in these patients.

### 3.4 TVRT Accelerates Improvement in Symptom Severity Scores in BPPV Patients With RD

To evaluate the efficacy of vestibular rehabilitation, the DHI and ABC scale were used to assess dizziness severity at multiple follow‐up time points. Both the control and tVRT groups showed progressive improvement over time, as reflected by decreased DHI scores (Table 1, Figure 2B) and increased ABC scores (Table 2, Figure 2C). Mixed‐effects model analysis demonstrated a significant main effect of time (both *p* < 0.0001) and a significant group × time interaction (DHI: *p* = 0.0017; ABC: *p* = 0.0008), whereas the main effect of group was not significant (DHI: *p* = 0.3633; ABC: *p* = 0.1587). Post hoc Tukey's within‐group comparisons confirmed significant improvements from baseline at all follow‐up time points within both groups (all adjusted *p* < 0.0001). Post hoc simple‐effects analysis with Šidák's multiple comparisons test showed that the tVRT group exhibited significantly lower DHI scores than the control group at week 4 (*p* = 0.0465), and significantly higher ABC scores at week 4 (*p* = 0.0164). The significant interaction indicates that the trajectories of recovery differed between the two groups, supporting a faster improvement in the tVRT group over time.

**TABLE 1:** Comparison of DHI scores in the two groups of patients at different periods.

Time points	DHI score	*p* value				
		Pre‐treatment	Post‐treatment (week)			
			1	2	3	4
***Ctrl group***						
Pre‐treatment	71.80 ± 17.67		^****^	^****^	^****^	^****^
1 week post‐treatment	41.00 ± 15.34			^****^	^****^	^****^
2 weeks post‐treatment	32.03 ± 13.85				^****^	^****^
3 weeks post‐treatment	21.07 ± 12.19					^****^
4 weeks post‐treatment	11.43 ± 7.20					
***tVRT group***						
Pre‐treatment	73.24 ± 16.64		^****^	^****^	^****^	^****^
1 week post‐treatment	40.67 ± 11.30			^****^	^****^	^****^
2 weeks post‐treatment	25.15 ± 10.00				^****^	^****^
3 weeks post‐treatment	17.73 ± 9.90					^****^
4 weeks post‐treatment	7.27 ± 5.28					

**** Means *p* < 0.0001.

**TABLE 2:** Comparison of ABC scores in the two groups of patients at different periods.

Time points	ABC score	p value				
		Pre‐treatment	Post‐treatment (week)			
			1	2	3	4
***Ctrl group***						
Pre‐treatment	33.50 ± 10.87		^****^	^****^	^****^	^****^
1 week post‐treatment	61.00 ± 13.48			^****^	^****^	^****^
2 weeks post‐treatment	73.53 ± 12.79				^****^	^****^
3 weeks post‐treatment	85.03 ± 9.56					^****^
4 weeks post‐treatment	90.60 ± 7.53					
***tVRT group***						
Pre‐treatment	32.76 ± 9.72		^****^	^****^	^****^	^****^
1 week post‐treatment	62.06 ± 12.78			^****^	^****^	^****^
2 weeks post‐treatment	80.76 ± 9.97				^****^	^****^
3 weeks post‐treatment	89.97 ± 7.19					^****^
4 weeks post‐treatment	94.88 ± 5.88					

**** Means *p* < 0.0001.

SAS scores (Table 3, Figure 2D) also decreased progressively following treatment in both groups. Mixed‐effects model analysis demonstrated a significant main effect of time (*p* < 0.0001) and a significant group × time interaction (*p* < 0.0001), whereas the main effect of group was not significant (*p* = 0.0804). Within‐group comparisons demonstrated significant reductions from baseline at all follow‐up time points in both groups (all adjusted *p* < 0.0001). Post hoc simple‐effects analysis with Šidák's multiple comparisons test demonstrated significantly lower SAS scores in the tVRT group than in the control group at week 4 (*p* = 0.0025). The significant interaction indicates that the reduction in anxiety symptoms occurred at different rates between the two rehabilitation strategies, with a more favorable recovery trajectory in the tVRT group.

**TABLE 3:** Comparison of SAS scores in the two groups of patients at different periods.

Time points	SAS score	*p* value				
		Pre‐treatment	Post‐treatment (week)			
			1	2	3	4
***Ctrl group***						
Pre‐treatment	72.60 ± 10.62		^****^	^****^	^****^	^****^
1 week post‐treatment	60.53 ± 11.44			^****^	^****^	^****^
2 weeks post‐treatment	52.03 ± 12.52				^****^	^****^
3 weeks post‐treatment	32.10 ± 10.84					^****^
4 weeks post‐treatment	24.33 ± 8.79					
***tVRT group***						
Pre‐treatment	71.27 ± 12.62		^****^	^****^	^****^	^****^
1 weeks post‐treatment	58.48 ± 13.25			^****^	^****^	^****^
2 weeks post‐treatment	45.58 ± 12.74				^****^	^****^
3 weeks post‐treatment	26.55 ± 12.10					^****^
4 weeks post‐treatment	13.97 ± 8.62					

**** Means *p* < 0.0001.

### 3.5 Rs‐fMRI Reveals Enhanced Brain Functional Activity Following tVRT in BPPV Patients With RD

A total of pre‐specified 60 patients consented to undergo rs‐fMRI before initiating vestibular rehabilitation. Pre‐treatment rs‐fMRI analysis revealed no significant differences in brain activity between the control and tVRT groups. After completing the 4‐week rehabilitation program, 17 patients failed to complete the post‐treatment brain imaging, resulting in 43 patients included in the final analysis—20 in the control group (8 males, 12 females) and 23 in the tVRT group (10 males, 13 females). The mean age of the control group was 49.29 ± 6.83 years, and that of the tVRT group was 47.64 ± 8.51 years, with no significant age difference between groups (Table 4). No significant differences were observed in age, gender, DHI, SAS, ABC scores, or oVEMP abnormality rate between the tVRT and control groups, confirming no selection bias in the fMRI subgroup (Table 4).

**TABLE 4:** Statistics on the general information of the two groups of rs‐fMRI patients.

Characteristic	Ctrl group (*n* = 20)	tVRT (*n* = 23)	*p* value
Age	49.29 ± 6.83	47.64 ± 8.51	0.47
Male, *n* (%)	8 (40%)	10 (43.5%)	0.82
DHI score	70.25 ± 16.82	72.13 ± 15.97	0.69
SAS score	71.85 ± 9.74	70.92 ± 11.36	0.78
ABC score	34.12 ± 10.23	33.47 ± 9.85	0.83
oVEMP abnormality rate, *n* (%)	20 (100%)	23 (100%)	1

**FIGURE 3:**
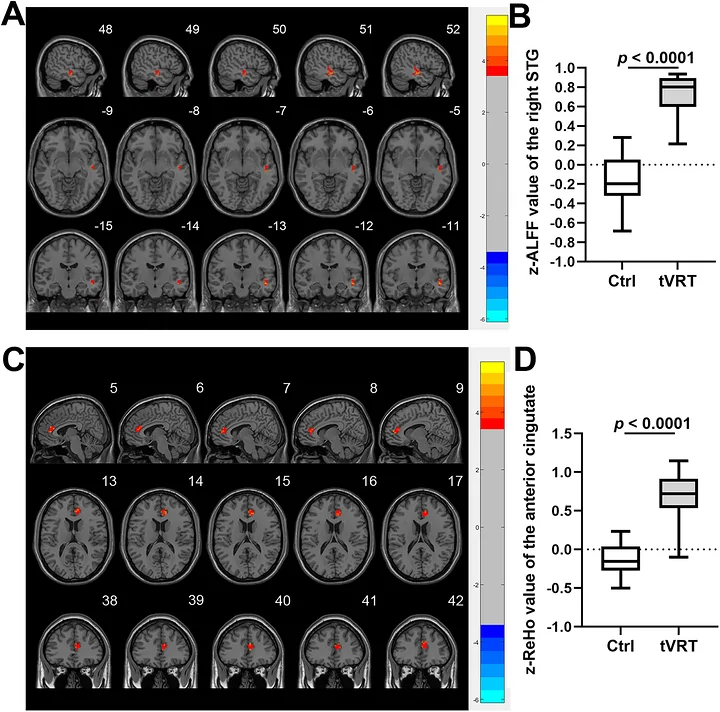
**Cerebral functional imaging in BPPV patients with RD after tVRT treatment**. (A) Compared to the conventional group, the ALFF value of the right superior temporal gyrus (STG) is increased in tVRT group in BPPV patients with RD. Red represents a significant increase in ALFF value. Red warm colors indicate increased ALFF, whereas blue cool colors indicate decreased ALFF. (B) The statistically results of z‐ALFF value of the right STG between the tVRT and conventional control group. (C) Compared to the conventional group, the ReHo value of the anterior cingulate is increased in tVRT group in BPPV patients with RD. Red represents a significant increase in ReHo value. Red warm colors indicate increased ReHo, whereas blue cool colors indicate decreased ReHo. (D) The statistically results of ReHo value of the anterior cingulate between the tVRT and conventional control group. Data are presented as mean ± SD, *t*‐test.

To investigate the effects of vestibular rehabilitation on brain functional activity in BPPV RD patients with isolated utricular dysfunction, we analyzed rs‐fMRI data collected after 4 weeks of treatment. The results (Table 5 and Figure 3A) demonstrated a significantly higher ALFF in the right superior temporal gyrus (STG) in the tVRT group compared to the control group (*p* < 0.0001, *t*  =  12.39, df  =  41), indicating enhanced neural activity in this region. Previous studies have shown that the STG plays a key role in the integration of vestibular and visual information (Ventre‐Dominey 2014). The increased ALFF in the right STG in the tVRT group may reflect more pronounced neuroplastic changes associated with visual compensation, suggesting more efficient vestibular‐visual integration in these patients.

**TABLE 5:** The brain regions showing significant differences in ALFF and ReHo values between two groups.

Brain region (AAL)	Number of voxels	MNI coordinates (*x*, *y*, *z*)	Peak *t* value
***ALFF values***			
Temporal_Sup_R	86	50, −13, −7	3.54
***ReHo values***			
Cingulum_Ant_R	143	7, 40, 15	3.32

As shown in Table 4 and Figure 3B, the ReHo value in the right anterior cingulate cortex (ACC) were significantly higher in patients receiving tVRT compared to those receiving control therapy (*p* < 0.0001, *t*  =  10.50, df  =  41), indicating that tVRT markedly enhances the local synchronization of neuronal activity in the right ACC. The ACC is a key node within both the default mode network and the salience network, and is critically involved in emotional and cognitive regulation (Wu et al. 2024). The increased ReHo in the right ACC observed in the tVRT group may reflect improved neural coordination associated with more efficient emotional and cognitive control mechanisms in these patients.

### 3.6 Rs‐fMRI Reveals Changed FC in Vestibular‐Related Networks Following tVRT in BPPV Patients With RD

**FIGURE 4:**
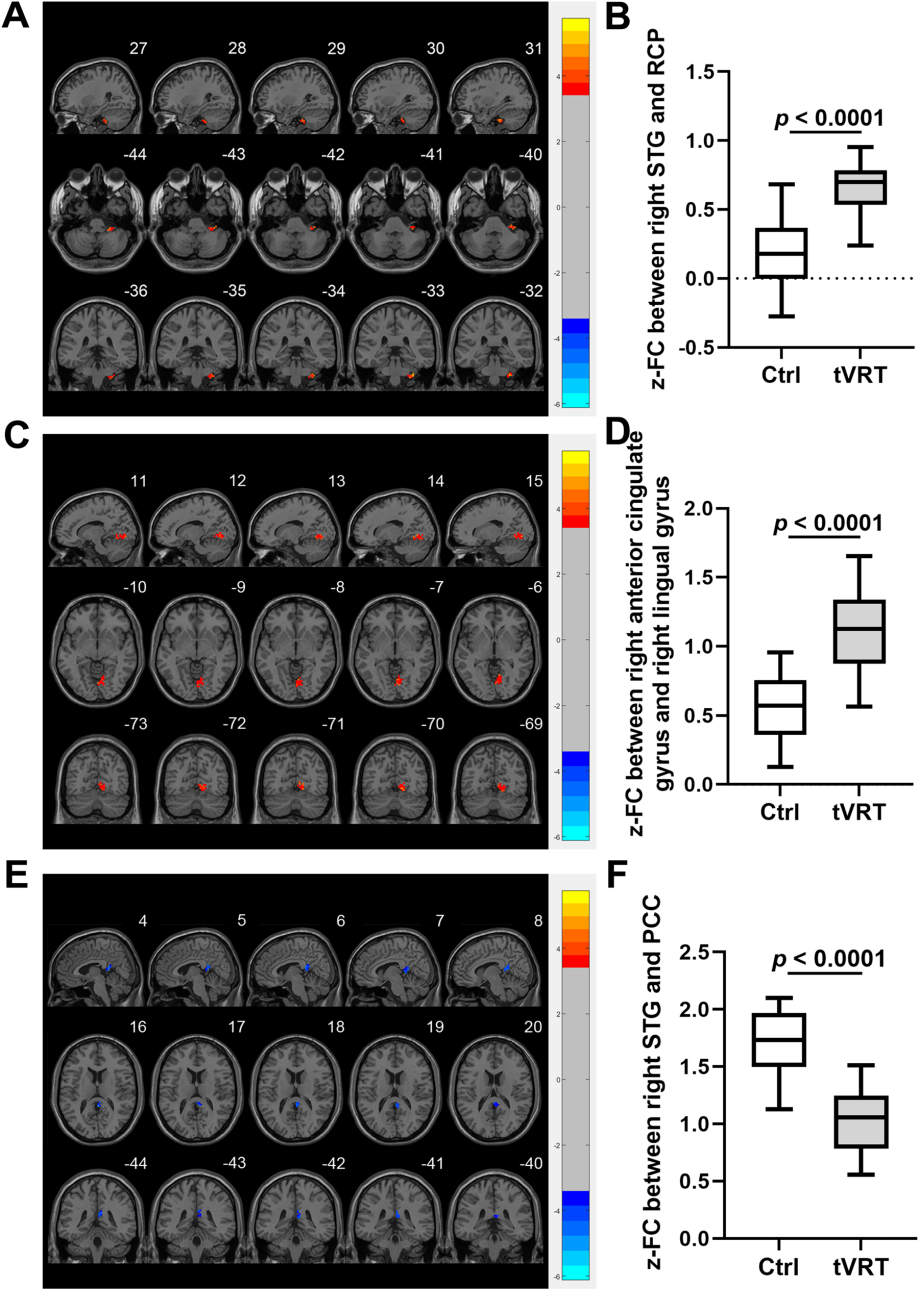
**Cerebral functional imaging in BPPV patients with RD after tVRT treatment**. (A, B) When the right STG was used as the seed region, after tVRT, BPPV patients with RD exhibited enhanced FC between the right STG and the RCP. (C, D) When the right ACC was used as the seed region, BPPV patients with RD after tVRT exhibited increased FC between the right ACC and the right lingual gyrus. (E, F) When the right STG was used as the seed region, BPPV patients with RD after tVRT exhibited reduced FC between the right STG and the right PCC. The color scale represents Z‐transformed FC values. Red and blue mean stronger and weaker connectivity, respectively. Data are presented as mean ± SD, *t*‐test.

To investigate the effect of tVRT on brain FC in BPPV RD patients with isolated utricular dysfunction, seed‐based FC analyses were performed using the right STG and ACC as seed regions. As shown in Table 6 and Figure 4, compared with the control group, patients in the tVRT group exhibited significantly enhanced FC between the right STG and the right cerebellar flocculus (*p* < 0.0001, *t*  =  7.132, df  =  41) (Figure 4A and B). This enhancement suggests a more efficient integration within the vestibulo‐cerebellar pathway in the tVRT group, potentially contributing to improved motor coordination, balance, spatial orientation, and postural control.

**TABLE 6:** The brain regions showing significant differences in FC between two groups.

ROI	Brain region (AAL)	Number of voxels	MNI coordinates (*x*, *y*, *z*)	Peak *t* value
Temporal_Sup_R	Cerebelum_10_R	33	29, −34, −42	3.26
	Cingulum_Post_R	64	6, ‐42, 18	−4.15
Cingulum_Ant_R	Lingual_R	138	13, ‐71, ‐8	4.7

Moreover, when the right ACC was used as the seed region, FC between the ACC and the lingual gyrus was significantly increased in the tVRT group (*p* < 0.0001, *t*  =  6.695, df  =  41) (Figure 4C and D). The ACC is involved in emotional regulation, while the lingual gyrus plays a role in visual information processing (Wu et al. 2024). This finding implies that tVRT may enhance the coordination between emotion‐ and vision‐related brain regions, which could facilitate more effective visual compensation and emotional regulation mechanisms in response to vestibular dysfunction.

Conversely, using the right STG as the seed region, the tVRT group showed significantly reduced FC between the right STG and the PCC (*p* < 0.0001, *t*  =  7.384, df  =  41) (Figure 4E and F). PCC is a core hub of the default mode network (DMN), and reduced FC within the DMN may reflect decreased pathological self‐referential processing, which has been associated with persistent dizziness symptoms (Klingner et al. 2014). Therefore, the weakened STG–PCC connectivity observed in the tVRT group may indicate a lower likelihood of developing chronic dizziness symptoms after treatment.

### 3.7 TVRT Alters Correlation Between Brain Function and Clinical Outcomes in BPPV RD Patients

**FIGURE 5:**
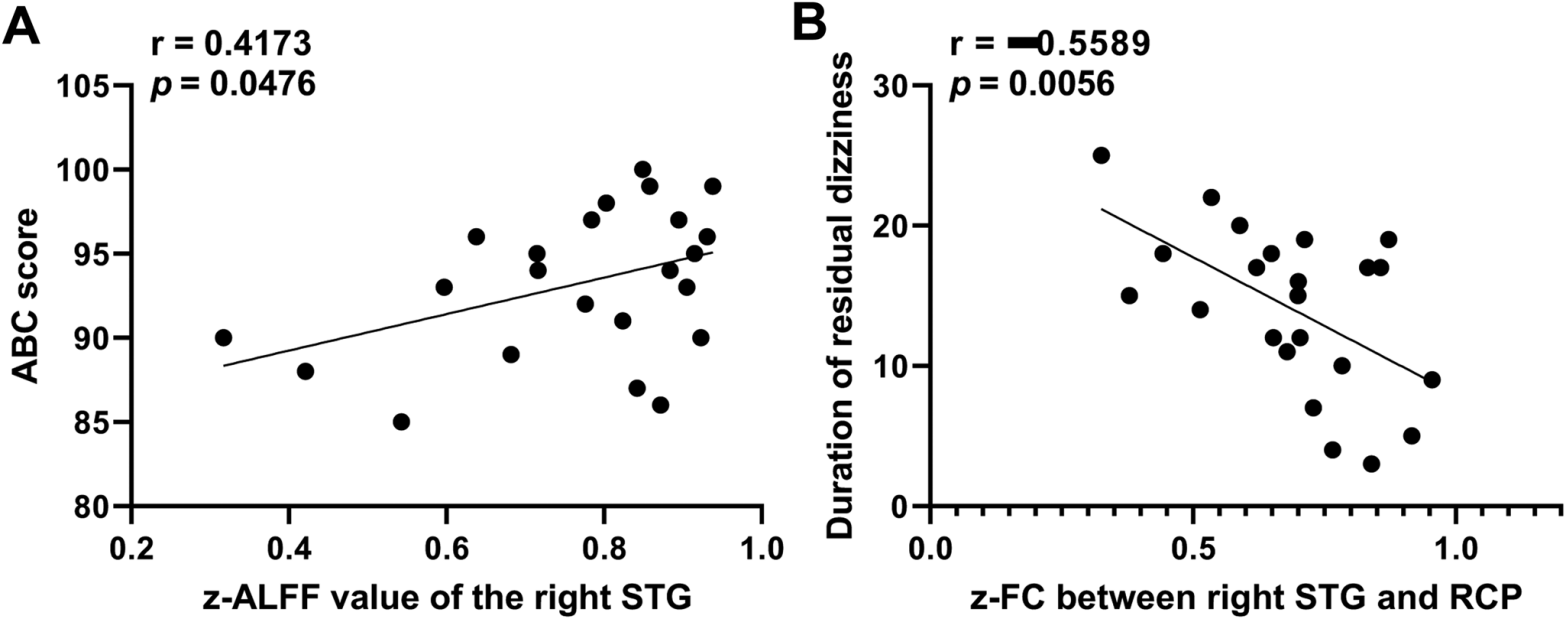
**tVRT alters correlation between brain function and clinical outcomes in BPPV patients with RD**. (A) The ALFF values in BPPV patients with RD in the tVRT group were positively correlated with ABC scores. (B) The FC between the right STG and the right cerebellar flocculus in BPPV patients with RD in the tVRT group was negatively correlated with the duration of RD.

To further explore the relationship between brain functional alterations and clinical improvements following tVRT therapy, correlation analyses were performed in 23 patients from the tVRT group after 4 weeks of therapy. As shown in Figure 5A, Pearson's correlation analysis showed that the z‐ALFF in the right STG was positively correlated with the ABC score (*p*  =  0.0476, *r*  =  0.4173). After adjustment for age and sex using partial correlation analysis, this association remained positive but was attenuated and no longer reached statistical significance (partial *p* = 0.0662, *r* = 0.389). This result suggests that stronger regional neural activity in the right STG is associated with better balance confidence in BPPV patients with RD.

Furthermore, the correlation between FC and RD symptom duration was analyzed. As shown in Figure 5B, the z‐FC value between the right STG and the right cerebellar tonsil was negatively correlated with the duration of RD following tVRT (*p*  =  0.0056, *r*  =  –0.5589). This negative correlation remained significant after adjustment for age and sex (partial *p* = 0.0054, *r* = −0.560). These findings indicate that enhanced FC within the vestibulo‐cerebellar circuit may facilitate vestibular compensation and thus shorten the duration of RD.

## 4 Discussion

Our findings indicate that while tVRT does not directly restore utricular function in BPPV RD patients with isolated utricular dysfunction, it significantly improves clinical outcomes, including reduced DHI and SAS scores and increased ABC scores. Notably, the rate of improvement in these clinical indicators was faster in the tVRT group compared to the conventional rehabilitation group. This suggests that tVRT may accelerate vestibular compensation, particularly in the early phase of recovery, by targeting the impaired utricle and promoting central adaptation. In the later stages, further integration of vestibulo‐ocular reflexes, vestibulospinal reflexes, and vestibulocollic reflexes may help re‐establish global postural and spatial equilibrium, maintaining therapeutic superiority throughout the course of rehabilitation. One of the major advantages of tVRT lies in its ability to deliver site‐specific rehabilitation training. Protocols can be individually tailored based on the type, location, and severity of vestibular dysfunction, which is critical as different vestibular disorders often require distinct therapeutic strategies to achieve optimal recovery outcomes (Jafarzadeh et al. 2018). Our study provides evidence that such targeted rehabilitation, compared with standard general protocols, may offer enhanced recovery by aligning more closely with the lesion‐specific rehabilitation needs and goals of each patient. Previous studies have demonstrated that several rehabilitation strategies, including virtual reality (VR)‐assisted rehabilitation, medication combined with rehabilitation, and individualized vestibular rehabilitation, can improve recovery in patients with vestibular disorders. For example, VR‐assisted rehabilitation primarily enhances multisensory integration and motor learning through immersive visual stimulation, whereas medication mainly provides symptomatic relief and facilitates participation in rehabilitation (Chen et al. 2012). However, few prior studies have explicitly designed rehabilitation protocols that specifically address the impaired vestibular structure. To partially address this gap, we discussed and contrasted personalized vestibular rehabilitation, which also aims to optimize training based on individual conditions. For example, Wang et al. reported that short‐term individualized vestibular rehabilitation training (consisting of a complex set of physical exercises) significantly improved multiple clinical outcomes—DHI, SAS, ABC, VAS, GAD‐7, UW, and vHIT scores—within just 2 weeks in BPPV patients (Wang et al. 2024). Similarly, To et al. confirmed that a 6‐week individualized vestibular rehabilitation program following canalith repositioning maneuver was more effective than repositioning alone in improving balance and gait in adults with unilateral posterior canal BPPV (Se To et al. 2022). These findings support the notion that personalized rehabilitation strategies can significantly accelerate vestibular compensation in BPPV. In contrast, tVRT differs from these approaches by specifically targeting the anatomically impaired vestibular structure. Rather than selecting exercises solely according to overall functional impairment, tVRT employs lesion‐specific rehabilitation protocols designed to stimulate the affected vestibular end organ, thereby potentially facilitating more efficient central adaptation. In summary, both previous research and our current study demonstrate that tVRT, as a lesion‐specific and precision‐oriented approach, may expedite central compensation and recovery in BPPV patients with isolated utricular dysfunction. As summarized in Table 7, tVRT is designed based on a clear diagnostic target—such as a specific type of vestibular lesion or symptom—and uses specialized exercises to improve that target function. These findings highlight the clinical value of targeted rehabilitation strategies in optimizing therapeutic efficacy and reducing recovery time in vestibular disorders.

**TABLE 7:** Comparison of conventional vestibular rehabilitation, individualized program focusing on body state, and targeted vestibular rehabilitation.

Types	Conventional rehabilitation	Personalized vestibular rehabilitation	tVRT
Efficacy onset speed	Slower	Faster	Faster
Whether it targets specific disease types	Yes	Yes	Yes
Whether it specifies the lesion site	No	Yes	Yes
Whether it considers individual needs and differences	No	Yes	No
Whether it focuses on specific training actions	No	Yes	Yes
Whether it needs to comprehensively consider the patient's overall condition	No	Yes	No
Whether it is easy to control and standardize the treatment protocol	Yes	No	Yes
Whether it allows for double‐blind comparison	Yes	No	Yes
Whether it allows for quantitative evaluation	Yes	No	Yes

Our rs‐fMRI findings provide novel insights into the central mechanisms by which tVRT may accelerate vestibular compensation in BPPV patients with RD due to isolated utricular dysfunction. The dissociation between unchanged oVEMP findings and improved clinical outcomes is consistent with the classical concept of vestibular compensation, in which functional recovery primarily depends on central neuroplasticity rather than restoration of peripheral vestibular end‐organ function. Because oVEMP reflects the integrity of the utricular macula and the superior vestibular nerve pathway, persistent oVEMP abnormalities suggest that the peripheral lesion itself remains largely unchanged. Vestibular rehabilitation is not expected to regenerate damaged utricular sensory receptors; instead, it promotes adaptation, sensory reweighting, and multisensory integration within the central vestibular network. Accordingly, although tVRT did not enhance the peripheral recovery of utricular function per se, it significantly altered local brain activity and FC. Importantly, these neural changes were closely associated with improvements in clinical outcomes, including reductions in dizziness and anxiety symptoms and improvements in balance confidence. These results suggest that tVRT facilitates recovery not by restoring peripheral vestibular function but by enhancing central compensation through neuroplastic reorganization. Specifically, rs‐fMRI analyses indicated that tVRT was associated with significant functional remodeling in cortical and subcortical vestibular‐related brain regions. These included increased regional neural activity and enhanced FC among key vestibular and visuospatial processing areas, implying a possible mechanism involving cortical and subcortical functional reorganization (Chen et al. 2024). For instance, patients receiving tVRT showed enhanced FC between the ACC and the lingual gyrus—a circuit potentially involved in anxiety‐visual compensation. The lingual gyrus plays a critical role in visual processing, while the ACC is implicated in emotional regulation; strengthened FC between these regions may reflect improved visuovestibular integration and compensatory adaptation for visual vertigo. These findings are consistent with previous studies demonstrating that vestibular rehabilitation after successful CRP can improve RD via enhanced connectivity among brain regions involved in vestibular processing and emotional regulation (Chen et al. 2025). Other studies have reported that vestibular rehabilitation increases neural activity in areas responsible for vestibulo‐visual interaction and integration (Wu et al. 2024), while changes in the subiculum (a hippocampal region critical for spatial orientation and mood) have also been observed in association with symptom improvement following vestibular rehabilitation (Chen et al. 2024). Furthermore, enhanced FC between the superior temporal gyrus and cerebellar flocculus in the tVRT group suggests a possible facilitation of the vestibulo‐cerebellar‐cortical integration loop. The cerebellar flocculus is a crucial relay in vestibular reflex pathways, and its interaction with the superior temporal gyrus may represent improved coordination between sensory perception and motor control systems. This observation supports the hypothesis that tVRT promotes not only localized cortical plasticity but also the strengthening of large‐scale brain networks involved in vestibular processing. Previous research has also demonstrated that vestibular rehabilitation can enhance connectivity within parietal and occipital regions, which are essential for the integration of visual and vestibular information (Chen et al. 2024). The consistency between our results and these studies suggests that tVRT may exert its therapeutic effects through promoting structural and functional reorganization within these distributed neural networks. Taken together, our findings support the hypothesis that the effectiveness of tVRT in alleviating RD of BPPV may be attributed to its ability to modulate FC and neural activity in specific brain regions involved in vestibular compensation. This neural remodeling includes both localized enhancements in activity and network‐level optimizations, ultimately facilitating more efficient central integration of vestibular inputs and thereby accelerating recovery.

**FIGURE 6:**
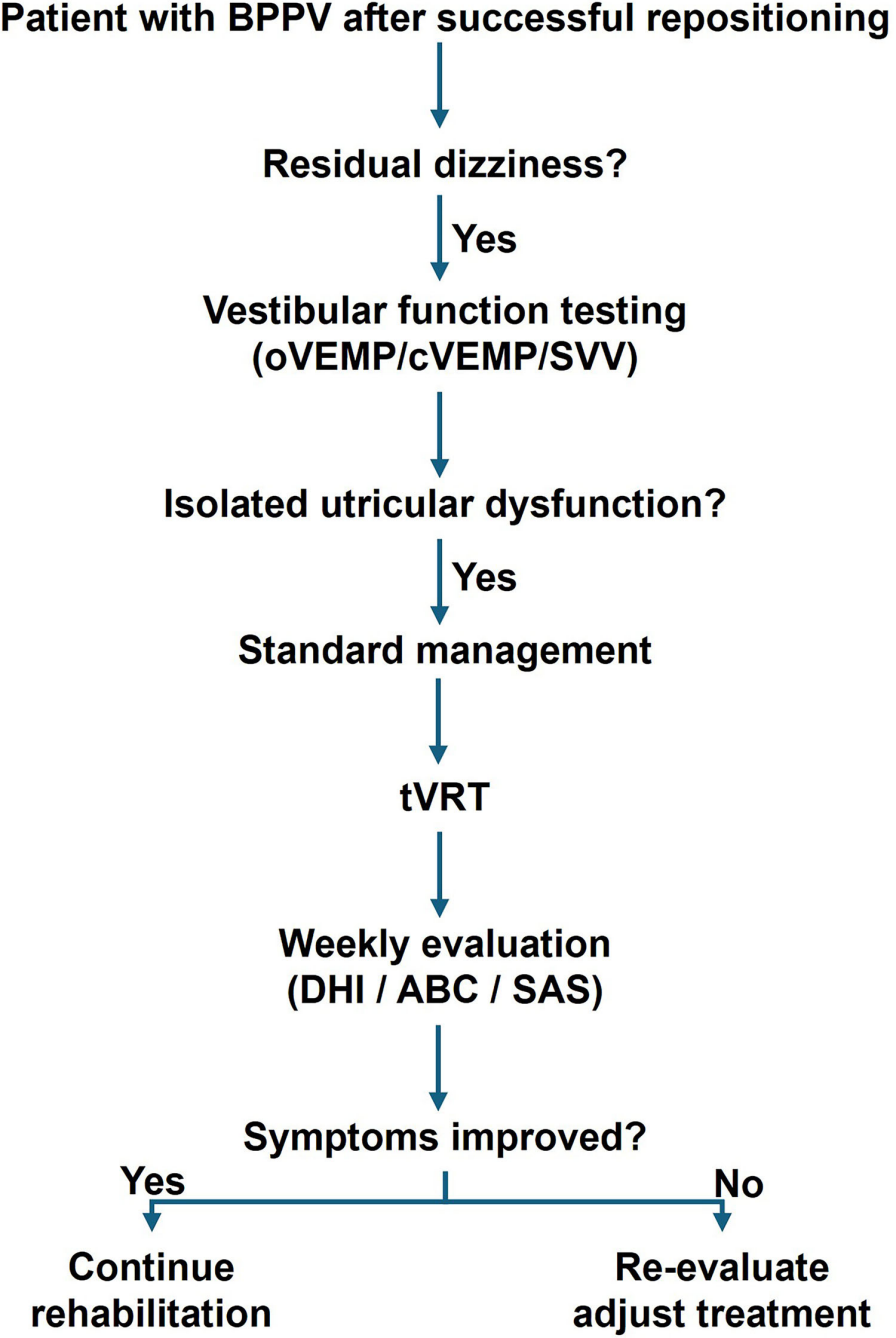
Proposed standardized clinical workflow for tVRT in patients with BPPV‐associated RD and isolated utricular dysfunction.

To facilitate the clinical translation of tVRT, we propose a standardized clinical workflow for patient selection and intervention (Figure 6). First, the diagnosis of BPPV should be confirmed following successful canalith repositioning, and patients with persistent positional rotational vertigo should be excluded to avoid including those with unsuccessful repositioning. Second, vestibular function testing, including oVEMP, cVEMP, and SVV, should be performed to identify patients with isolated utricular dysfunction who are most likely to benefit from tVRT. Third, eligible patients should receive standardized medical treatment together with either conventional vestibular rehabilitation or tVRT during hospitalization (5–7 days). Fourth, after discharge, patients should continue the prescribed home‐based rehabilitation program and undergo scheduled follow‐up assessments at 1, 2, 3, and 4 weeks to evaluate vestibular function and clinical outcomes. Finally, rehabilitation should be discontinued if severe adverse events occur, whereas patients with recurrent or worsening symptoms should undergo comprehensive re‐evaluation and receive appropriate treatment adjustments. This standardized workflow provides a practical framework for implementing tVRT in clinical practice and may facilitate its broader application in patients with BPPV‐associated RD.

This study has several limitations. First, it was conducted at a single center, which may limit the generalizability of the findings. Second, only patients aged 18–60 years were included; therefore, the efficacy of tVRT in older adults, who represent a substantial proportion of patients with BPPV, remains to be determined. Third, the follow‐up period was limited to 4 weeks, precluding evaluation of the long‐term effects of tVRT on vestibular compensation and recurrence. Finally, adherence to the prescribed home‐based rehabilitation program after discharge was not objectively monitored, which may have introduced variability in treatment compliance. Future multicenter studies with broader age inclusion, longer follow‐up, and objective adherence monitoring are warranted to further validate our findings.

## 5 Conclusion

tVRT, based on conventional vestibular rehabilitation principles, does not restore utricular function in BPPV RD patients but significantly accelerates clinical recovery and improves quality of life. rs‐fMRI findings suggest that the therapeutic effects of tVRT are mediated by modulation of neural activity and optimization of functional connectivity within key vestibular‐related brain regions. These central adaptations may facilitate vestibular compensation, thereby alleviating RD more effectively than conventional rehabilitation. Collectively, based on the current findings, tVRT may be considered for adult patients with BPPV‐associated RD and confirmed isolated utricular dysfunction following successful CRP. A 4‐week tVRT program consisting of three daily sessions with 30 repetitions per exercise, combined with conventional VRT, appears to provide effective clinical benefits. However, these recommendations should be interpreted cautiously until validated in larger multicenter studies.

## Author Contributions

**Lingwei Li**: formal analysis, investigation, software, visualization. **Xiangli Zeng**: conceptualization, project administration, resources, supervision, writing – review and editing. **Chang Liu**: conceptualization, project administration, resources, supervision, writing – review and editing. **Gendi Yin**: conceptualization, data curation, funding acquisition, methodology, visualization, writing – original draft. **Jiashu Lin**: formal analysis, investigation, methodology, validation. **Zhicheng Li**: conceptualization, investigation, project administration, resources, supervision, writing – review and editing. **Qiling Huang**: data curation, formal analysis, methodology, validation, writing – original draft. **Yinfei Liang**: data curation, writing – original draft.

## Funding

This work was supported by grants from the National Natural Science Foundation of China (82301312, 82171151); 2025 Space Medicine Fund of The Third Affiliated Hospital of Sun Yat‐sen University (2025TKYXMS01); Research Project of the Traditional Chinese Medicine Bureau of Guangdong Province (Grant No. 20231065) and Teaching Reform Project of the New Medical Education Teaching Steering Committee of Guangdong Province (Grant No. GD‐NME2023‐055).

## Ethics Statement

This study was approved by the Institutional Review Board of Third Affiliated Hospital of Sun Yat‐Sen University (Approval No. II2025‐527‐01).

## Patient Consent Statement

All participants provided written informed consent prior to participation in this study.

## Conflicts of Interest

The authors declare that there are no conflicts of interest.

## Supporting information



Table S1 Statistics on the general information of the two groups of patients.
Table S2 Comparison of oVEMP changes in the two groups of patients after treatment.
Table S3 Comparison of vHIT and CT changes in the two groups of patients after treatment.

## References

[brb371696-bib-0001] Bhattacharyya, N. , S. P. Gubbels , S. R. Schwartz , et al. 2017. “Clinical Practice Guideline: Benign Paroxysmal Positional Vertigo (Update).” Otolaryngology–Head and Neck Surgery: Official Journal of American Academy of Otolaryngology‐Head and Neck Surgery 156, no. 3: S1–s47. 10.1177/0194599816689667 28248609

[brb371696-bib-0002] Chen, P. Y. , W. L. Hsieh , S. H. Wei , and C. L. Kao . 2012. “Interactive Wiimote Gaze Stabilization Exercise Training System for Patients With Vestibular Hypofunction.” Journal of NeuroEngineering and Rehabilitation 9: 77. 10.1186/1743-0003-9-77. 23043886 PMC3481473

[brb371696-bib-0003] Chen, Z. , Y. Cai , Y. Liu , et al. 2024. “Altered Thalamus Functional Connectivity in Patients With Acute Unilateral Vestibulopathy: A Resting‐State fMRI Study.” Frontiers in Neuroscience 18: 1388213. 10.3389/fnins.2024.1388213. 39010942 PMC11246849

[brb371696-bib-0004] Chen, Z. , Y. Liu , C. Lin , et al. 2024. “Altered Parietal Operculum Cortex 2 Functional Connectivity in Benign Paroxysmal Positional Vertigo Patients With Residual Dizziness: a Resting‐State fMRI Study.” CNS Neuroscience & Therapeutics 30, no. 2:e14570. 10.1111/cns.14570. 38421104 PMC10850607

[brb371696-bib-0005] Chen, Z. , Y. Liu , Y. Sun , et al. 2025. “Increased Parietal Operculum Functional Connectivity Following Vestibular Rehabilitation in Benign Paroxysmal Positional vertigo Patients With Residual Dizziness: A Randomized Controlled Resting‐State fMRI Study.” Neuroradiology 67, no. 4: 931–942. 10.1007/s00234-024-03535-4. 39754615

[brb371696-bib-0006] Chen, Z. , L. Xiao , Y. Liu , et al. 2024. “Altered Hippocampal Subfields Functional Connectivity in Benign Paroxysmal Positional Vertigo Patients With Residual Dizziness: A Resting‐State fMRI Study.” CNS Neuroscience & Therapeutics 30, no. 12: e70175. 10.1111/cns.70175. 39690894 PMC11652785

[brb371696-bib-0007] Hillier, S. L. , and M. McDonnell . 2011. “Vestibular Rehabilitation for Unilateral Peripheral Vestibular Dysfunction.” Cochrane Database of Systematic Reviews 1, no. 2: CD005397. 10.1002/14651858.CD005397.pub3 21328277

[brb371696-bib-0008] Hoseinabadi, R. , A. Pourbakht , N. Yazdani , et al. 2018. “The Effects of the Vestibular Rehabilitation on the Benign Paroxysmal Positional Vertigo Recurrence Rate in Patients With Otolith Dysfunction.” Journal of Audiology and Otology 22, no. 4: 204–208. 10.7874/jao.2018.00087. 30016856 PMC6233938

[brb371696-bib-0009] Jafarzadeh, S. , A. Pourbakht , E. Bahrami , S. Jalaie , and A. Bayat . 2018. “Effect of Early Vestibular Rehabilitation on Vertigo and Unsteadiness in Patients With Acute and Sub‐Acute Head Trauma.” Iranian Journal of Otorhinolaryngology 30, no. 97: 85–90. 29594074 PMC5866486

[brb371696-bib-0010] Kim, H. J. , J. Park , and J. S. Kim . 2021. “Update on Benign Paroxysmal Positional Vertigo.” Journal of Neurology 268, no. 5: 1995–2000. 10.1007/s00415-020-10314-7. 33231724 PMC7684151

[brb371696-bib-0011] Klingner, C. M. , G. F. Volk , S. Brodoehl , O. W. Witte , and O. Guntinas‐Lichius . 2014. “Disrupted Functional Connectivity of the Default Mode Network due to Acute Vestibular Deficit.” NeuroImage: Clinical 6: 109–114. 10.1016/j.nicl.2014.08.022. 25379422 PMC4215422

[brb371696-bib-0012] Kunelskaya, N. L. , A. L. Guseva , E. A. Manaenkova , M. A. Chugunova , and Z. O. Zaoeva . 2023. “Vestibular Evoked Myogenic Potentials in Patients With Recurrent Benign Paroxysmal Positional Vertigo.” Russian Bulletin of Otorhinolaryngology 88, no. 2: 4. 10.17116/otorino2022880214. 37184547

[brb371696-bib-0013] Lacour, M. , C. Helmchen , and P. P. Vidal . 2016. “Vestibular Compensation: The Neuro‐Otologist's Best Friend.” Journal of Neurology 263, no. 1: S54–64. 10.1007/s00415-015-7903-4. 27083885 PMC4833803

[brb371696-bib-0014] López‐Viñas, L. , E. Rocío‐Martín , E. D.e L.a R. Santiago , J. P. Pendolero , and R. Wix‐Ramos . 2024. “Diagnostic Value of Vestibular Evoked Myogenic Potentials in Benign Paroxysmal Positional vertigo.” Acta Otorrinolaringológica Española 75, no. 3: 192–196. 10.1016/j.otorri.2023.10.006. 38220052

[brb371696-bib-0015] Niu, X. , P. Han , M. Duan , et al. 2022. “Bilateral Dysfunction of Otolith Pathway in Patients With Unilateral Idiopathic BPPV Detected by ACS‐VEMPs.” Frontiers in Neurology 13: 921133. 10.3389/fneur.2022.921133. 36090849 PMC9462380

[brb371696-bib-0016] Özgirgin, O. N. , H. Kingma , L. Manzari , and M. Lacour . 2024. “Residual Dizziness After BPPV Management: Exploring Pathophysiology and Treatment Beyond Canalith Repositioning Maneuvers.” Frontiers in Neurology 15: 1382196. 10.3389/fneur.2024.1382196. 38854956 PMC11157684

[brb371696-bib-0017] Rosa, M. S. , M. Campagnoli , D. Masnaghetti , et al. 2023. “Clinical and Prognostic Implications of Cervical and Ocular Vestibular Evoked Myogenic Potentials (cVEMP and oVEMP) in Benign Paroxysmal Positional Vertigo (BPPV): A Prospective Study.” Audiology Research 13, no. 5: 700–709. 10.3390/audiolres13050061. 37736942 PMC10514798

[brb371696-bib-0018] Seo, T. , K.o Shiraishi , T. Kobayashi , et al. 2017. “Residual Dizziness After Successful Treatment of Idiopathic Benign Paroxysmal Positional Vertigo Originates From Persistent Utricular Dysfunction.” Acta Oto‐Laryngologica 137, no. 11: 1149–1152. 10.1080/00016489.2017.1347824. 28681630

[brb371696-bib-0019] Se To, P. L. , D. K. A. Singh , and S. L. Whitney . 2022. “Effects of Customized Vestibular Rehabilitation plus Canalith Repositioning Maneuver on Gait and Balance in Adults With Benign Paroxysmal Positional Vertigo: A Randomized Controlled Trial.” Journal of Vestibular Research 32: 79–86. 34151874 10.3233/VES-190731

[brb371696-bib-0020] Vaduva, C. , J. Estéban‐Sánchez , R. Sanz‐Fernández , and E. Martín‐Sanz . 2018. “Prevalence and Management of Post‐BPPV Residual Symptoms.” European Archives of Oto‐Rhino‐Laryngology 275, no. 6: 1429–1437. 10.1007/s00405-018-4980-x. 29687182

[brb371696-bib-0021] Ventre‐Dominey, J. 2014. “Vestibular Function in the Temporal and Parietal Cortex: Distinct Velocity and Inertial Processing Pathways.” Frontiers in Integrative Neuroscience 8: 53. 10.3389/fnint.2014.00053. 25071481 PMC4082317

[brb371696-bib-0022] Wang, J. , Y. Lei , L. Tian , J. Zuo , Y. Shen , and J. Wang . 2024. “Application of Clinical Indicators in Evaluating Vestibular Compensation Efficacy in Benign Recurrent Vestibular Vertigo Patients With Short‐Term Personalized Vestibular Rehabilitation.” European Archives of Oto‐Rhino‐Laryngology 281, no. 7: 3509–3520. 10.1007/s00405-024-08457-8. 38261016 PMC11211146

[brb371696-bib-0023] Wu, J. , L. Shu , C. Y. Zhou , et al. 2024. “Brain Functional Alterations in Patients with Benign Paroxysmal Positional Vertigo Demonstrate the Visual–Vestibular Interaction and Integration.” Brain and Behavior 14, no. 10: e70053. 10.1002/brb3.70053. 39350430 PMC11442312

