# Supplementary material for: Targeted Vestibular Rehabilitation Accelerates Symptom Relief by Promoting Vestibular Compensation in BPPV Patients With Residual Symptoms: Evidence From Resting‐State fMRI
Source: Brain Behav. 2026 Aug 20;16(8):e71696. doi: 10.1002/brb3.71696 (PMC13494513; doi:10.1002/brb3.71696)
Supplement: Supplementary file 1 [file BRB3-16-e71696-s001.docx]

Table S1 Statistics on the general information of the two groups of patients

|  | Ctrl group | tVRT group | *p* value |
| --- | --- | --- | --- |
| Total | 45 | 49 | > 0.05 |
| Sex, n (%) |  |  |  |
| M | 23 (51.1%) | 25 (55.7%) | > 0.05 |
| F | 22 (48.9%) | 24 (53.3%) |  |
| Age | 47.33±8.72 | 48.09±10.20 | > 0.05 |
| DHI | 71.80±17.67 | 73.24±16.64 | > 0.05 |
| SAS | 72.60±10.62 | 72.27±12.62 | > 0.05 |
| ABC | 33.50±10.87 | 32.76±9.72 | > 0.05 |

Table S2 Comparison of oVEMP changes in the two groups of patients after treatment

| Group | Pre-treatment | | Post-treatment (week) | | | | | | | | | | | | | |
| --- | --- | --- | --- | --- | --- | --- | --- | --- | --- | --- | --- | --- | --- | --- | --- | --- |
|  |  |  | 1 | | | 2 | | | | 3 | | | | 4 | | |
|  | N | Abn | N | Abn | N | | | Abn | N | | Abn | | N | | Abn | |
| ***cVEMP*** |  |  |  |  |  | | |  |  | |  | |  | |  | |
| Ctrl group | 45 | 0 (0%) | 44 | 1 (2.2%) | 43 | | | 2 (4.4%) | 44 | | 1 (2.2%) | | 44 | | 1 (2.2%) | |
| tVRT group | 49 | 0 (0%) | 49 | 0 (0%) | 47 | | | 2 (4.1%) | 48 | | 1 (2.0%) | | 49 | | 0 (0%) | |
| *p* value | >0.05 | | >0.05 | | >0.05 | | | | >0.05 | | | | >0.05 | | | |
| ***oVEMP*** |  | |  | |  | | | |  | | | |  | | | |
| Ctrl group | 0 | 45 (100%) | 0 | 45 (100%) | 2 | | 43 (95.6%) | | 4 | | | 41 (91.1%) | 5 | | | 40 (88.9%) |
| tVRT group | 0 | 49 (100%) | 1 | 48 (98.0%) | 3 | | 46 (93.9%) | | 6 | | | 43 (87.8%) | 6 | | | 43 (87.8%) |
| *p* value | >0.05 | | >0.05 | | >0.05 | | | | >0.05 | | | | >0.05 | | | |

N means the count of individuals with normal VEMP elicitation, while Abn represents those with abnormal elicitation.

Table S3 Comparison of vHIT and CT changes in the two groups of patients after treatment

| Group | Pre-treatment | | Post-treatment (week) | | | | | | | | | | |
| --- | --- | --- | --- | --- | --- | --- | --- | --- | --- | --- | --- | --- | --- |
|  |  |  | 1 | | 2 | | | 3 | | | 4 | | |
|  | N | Abn | N | Abn | | N | Abn | | N | Abn | | N | Abn |
| *vHIT* |  |  |  |  | |  |  | |  |  | |  |  |
| Ctrl group | 45 | 0 (0%) | 45 | 0 (0%) | | 44 | 1 (2.2%) | | 45 | 0 (0%) | | 45 | 0 (0%) |
| tVRT group | 49 | 0 (0%) | 49 | 0 (0%) | | 49 | 0 (0%) | | 49 | 0 (0%) | | 48 | 1 (2.0%) |
| *p*value | > 0.05 | | > 0.05 | | | > 0.05 | | | > 0.05 | | | > 0.05 | |
| *CT* |  |  |  |  | |  |  | |  |  | |  |  |
| Ctrl group | 45 | 0 (0%) | - | - | | - | - | | - | - | | - | - |
| tVRT group | 49 | 0 (0%) | - | - | | - | - | | - | - | | - | - |
| *p*value | > 0.05 | | - | | | - | | | - | | | - | |

N means the count of individuals with normal VEMP elicitation, while Abn represents those with abnormal elicitation.
